# Floral Development on *Vitis vinifera* Is Associated with MADS-Box Transcription Factors through the Transcriptional Regulation of *VviZIP3*

**DOI:** 10.3390/plants12183322

**Published:** 2023-09-20

**Authors:** Germán Saavedra Núñez, Enrique González-Villanueva, Patricio Ramos

**Affiliations:** 1 Instituto de Ciencias Biológicas, Universidad de Talca, Talca 3460787, Chile; gesaavedra@utalca.cl (G.S.N.); egonzale@utalca.cl (E.G.-V.); 2 Vicerrectoría de Investigación y Postgrado, Universidad Católica del Maule, Talca 3480112, Chile

**Keywords:** grapevine, parthenocarpy, floral development, zinc transporter, transcriptional regulation

## Abstract

Several grapevine (*Vitis vinifera* L.) cultivars show a tendency to develop parthenocarpic seedless grapes, affecting fruit yield and quality. This reproductive disorder originates in defective ovule fertilization due to a failure in pollen tube growth. Zinc (Zn) is a crucial trace element, playing a vital role in various physiological and metabolic processes. It is particularly essential for the healthy growth of flowers and fruits. Insufficient zinc has been suggested as a potential reason for issues in this development process. This microelement is taken up through a mechanism that involves transporters, including the ZRT-IRT-like protein (ZIP) gene family, associated with the influx of Zn into the cell. In grapevines, 20 genes for ZIP-type transporters have been described. In this study, we analyzed the expression pattern of *VviZIP3* during flower development and employ transgenic methods to assess its transcriptional regulation. Furthermore, through computational examination of the promoter region, we identified two CArG boxes, recognized as responsive elements to MADS transcription factors. These factors play a key role in shaping various components of a flower, such as pollen. Our investigation of the *VviZIP3* promoter confirms the functionality of these CArG boxes. Overall, our results suggest that the increased expression of *VviZIP3* during flowering is likely under the influence of MADS transcription factors.

## 1. Introduction

Zinc (Zn) is an essential element for all organisms. It is the most abundant transition metal in living organisms after iron (Fe) and is the only metal present in all six classes of enzymes (oxidoreductases, transferases, hydrolases, lyases, isomerases, and ligases) [1]. Its role as a cofactor reaches more than 300 enzymes and proteins that participate in biological processes such as cell division, metabolism, protein synthesis, and regulation of gene transcription, being a cofactor of proteins that contain DNA-binding motifs of the Zn-finger type (“Zn-finger”), Zn ring (“RING-finger”), and LIM domains [1].

In grapevines, like most plants, Zn is beneficial in a narrow range of concentrations, and its bioavailability in soils increases at low pH [2,3]. The general effects of Zn phytotoxicity in plants are a reduction in root growth and stunted shoot growth [4,5]. In grapevines, the highest toxicity effect observed is a decrease in the root growth, but not at the level of the shoots, suggesting that shoots of the grapevines are more tolerant to exposure to Zn than the roots [6,7]. This effect of greater tolerance in the aerial tissue is short-term, but in the long term, greater negative effects could be evidenced.

Although the greatest impact of Zn deficiency is seen in the leaves, it has been observed that this deficiency can also lead to poor fruit sets and clusters containing small, green, and immature berries generated by a parthenocarpy event [8]. This phenomenon, also known as “millerandage”, can occur in all varieties. The extension of the “millerandage” is highly dependent on the variety analyzed; in this way, varieties with high and low tendency to “millerandage” can be defined [9], which suggests a specific genetic condition and reaction to the environmental conditions for each of them.

As the “millerandage” causes a decrease in yield, it becomes a problem for viticulture [10]. In view of this problem, various reasons have been sought to explain this phenomenon. A high tendency to “millerandage” has been associated with the development of abnormal pollen [11]. Several species possess various transcription factors with “Zn-finger” motifs that would be involved in pollen development and pollen tube growth [12,13], such as *Petunia hybrida* [14,15], *A. thaliana* [16], *Vitis vinifera* [17], *Triticum aestivum* [6], and *Zea mays* [18]. Hence, in the presence of a zinc deficiency within the plant, the proper formation of various “Zn-finger” transcription factors crucial for pollen development would be compromised. As a result, an anomalous pollen development would ensue, leading to the subsequent occurrence of parthenocarpic berries. In addition to the transcription factors that participate in pollen formation, other “Zn-finger”-type transcription factors participate in flower development. There are a diversity of examples, some of them are RABBIT EARS (RBE) [19], which participate in the development of petals, and KNUCKLES [20] and SUPERMAN [18], which participate specifically in the development of stamens and carpels; therefore, Zn deficiency could lead to an inadequate development of floral structures and, consequently, not forming berries or forming parthenocarpic berries.

The ZIP family is a broad group of membrane transporters that play an important role in the transport of Zn, Fe, Mn, Ni, Cu, and the toxic Cd [21]. As general characteristics, ZIP transporters have eight transmembrane domains, with their amino and carboxyl terminals oriented towards the outer surface of the plasma membrane [22]. Most of the ZIP proteins characterized in plants are located in the plasma membrane; however, some of them are found in the tonoplast or endomembrane system. Therefore, ZIP proteins would be involved in the regulation of uptake, accumulation, and translocation throughout the plant body, both of micronutrients and toxic heavy metals [23,24].

The ZIP family has been investigated in various species, and in several of them, the number of members has been identified. Thus, in *A. thaliana*, 15 have been identified [25], 23 in *Phaseolus vulgaris* [26], 12 in *Poncirus trifoliata* [27], 15 in *Oryza sativa* [28], 14 in *Triticum aestivum* [29], 12 in *Zea mays* [30], and 20 in *V. vinifera* [31].

Gainza-Cortés et al. [32] characterized one ZIP transporter of *V. vinifera*, which was named VvZIP3 and later modified to VviZIP3 according to the nomenclature of Grimplet et al. [33]. The authors suggested that *VviZIP3* encodes a high-affinity Zn uptake transporter in grapevines, and their higher expression is observed in reproductive tissues at the early stage of development. It was proposed that an increase in the expression of *VviZIP3* is necessary for the normal development of the flowers and berries of grapevines and that this increase coincides with the stages in which a large amount of Zn is required for flowering and fruit development [32].

On the other hand, genes that regulate flowering have been characterized in grapevine, including those for flower induction, flower meristem identity genes, and flower organ identity genes [34]. Within the identity genes of floral organs, the orthologs *AGAMOUS* and *APETALA1* to *A. thaliana* have been identified, called *VvMADS1* [35] and *VAP1* [36,37] and other *MADS* genes; therefore, it is possible to speculate that the flowering regulation mechanism in grapevines is similar to the ABCE model of *A. thaliana*.

Here, we report the analysis of the *VviZIP3* promoter, a member of the ZIP gene family from *V. vinifera* L. cv. Carménère, which encodes a Zn uptake protein. The mechanisms that regulate the expression of *VviZIP3* are still unknown. By employing a combination of computational, transcriptional, and transgenic approaches, we thoroughly evaluated the regulatory role of MADS-box transcription factors in mediating the transcriptional control of the *VviZIP3* promoter.

## 2. Results

### 2.1. Identification of MADS-Type Response Elements in VviZIP3 Promoter

**Figure 1:**
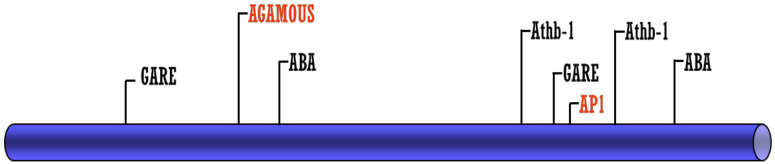
Graphic view of *VviZIP3* promoter and the main response elements found in the *VviZIP3* promoter. The response elements to floral organ identity genes are highlighted in red.

In order to understand the possible mechanism that regulates the expression of the *VviZIP3* gene, a first approach was made by performing an in silico analysis of the promoter region using the software JASPAR 2018 [38] and ConSite [39]. The analysis yielded a large number and diversity of response elements associated with signal transduction pathways related to plant hormones, such as auxins, gibberellins (GA), and abscisic acid (ABA) (Figure 1 and Table 1).

The analysis also showed the presence of two response elements called CArG box related to “floral organ identity genes”, both called AGAMOUS (AG) and APETALA1 (AP1) in *A. thaliana*, whose respective transcription factors belong to the MADS family (Figure 1). The same in silico analysis was performed on promoters of other members of the ZIP family of grapevines and, interestingly, these types of elements were only found in *VviZIP3*.

**Figure 2:**
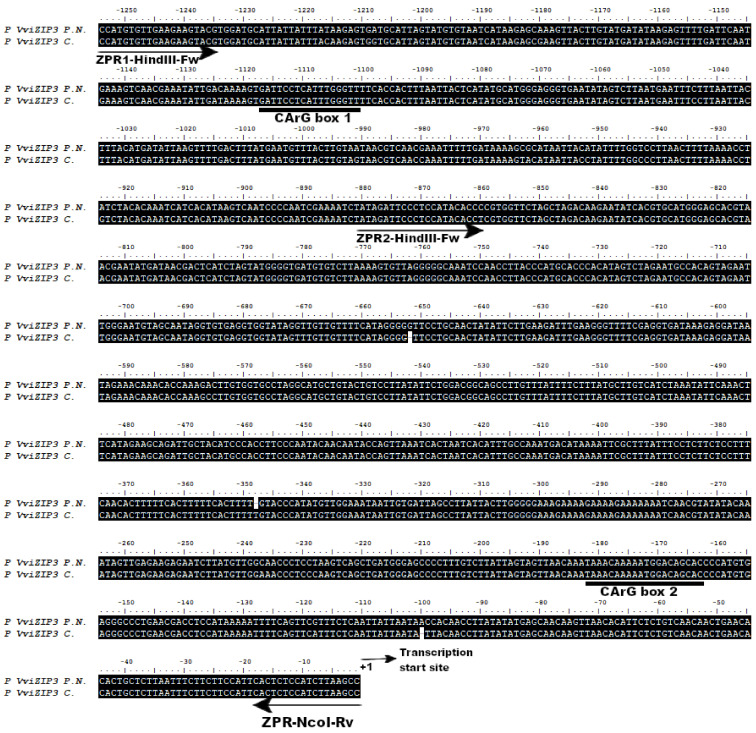
Comparison between nucleotide sequences of *VviZIP3* promoter in Pinot Noir (P.N) and Carmenérè (C). Identical amino acids are highlighted in black, dashes are deletions or insertions, and the location of the CArG boxes are underlined with black lines under the sequences. Black arrows represent ZPR primers. ZPR1-HindIII-Fw and ZPR-NcoI-Rv amplify the PZIP3Δ1 fragment. ZPR2-HindIII-Fw and ZPR-NcoI-Rv amplify the PZIP3Δ2 fragment. The transcription start site is labeled.

The two putative CArG box sequences, called CArG box 1 (AG) and CArG box 2 (AP1), were analyzed and compared with the CC(A/T)6GG consensus sequence identified by Shore and Sharrocks [40] (Table 1 and Figure 2).

The CArG box 1 site is 1115 bp from the translation start site and the CArG box 2 site is 181 bp from the translation start site. The sequences of CArG box 1 and 2 predicted by the search systems correspond to GATTCCTCATTTGGGTT and TAAACAAAAATGGACAGCAC, respectively (Table 1).

### 2.2. Isolation of Promoter Region of VviZIP3 in Carmenérè

Once the position of both CArG boxes has been identified, ZPR1 primers were designed, which allowed us to amplify the promoter region of *VviZIP3* in cv. Carmenérè, whose sequence was confirmed via sequencing. The sequences of cv. Pinot Noir and Carmenérè are presented in Figure 2. The sequence information was used to design the primers to amplify the full promoter and to design the truncated promoter constructions.

### 2.3. The MADS AGAMOUS and APETALA1 Proteins Belonging to V. vinifera Are Similar to Their Orthologs in A. thaliana

*A. thaliana* plants were transformed with the *VviZIP3* promoter. That is why it was necessary to analyze whether there is a similarity between MADS proteins AG and AP1 of *V. vinifera*, with MADS proteins AG and AP1 of *A. thaliana*.

A phylogenetic analysis was performed by Diaz-Riquelme et al. [41] to examine relationships among full-length grapevine MIKC proteins, including AG and AP1. This association demonstrates the closeness between AG of *V. vinifera* and AG of *A. thaliana* as well as between AP1 of *V. vinifera* and AP1 of *A. thaliana*.

### 2.4. Functionality of CArG Boxes Present in VviZIP3 Promoter

**Figure 3:**
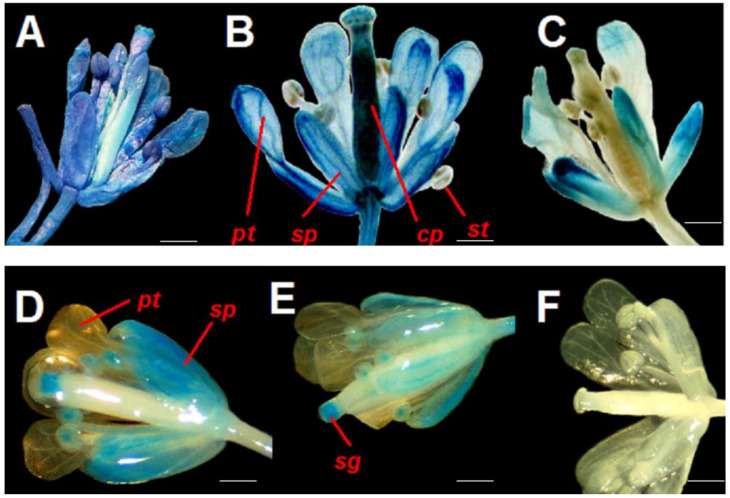
GUS histochemical staining in *A. thaliana* flowers containing the fragments. (**A**) *p35S*; (**B**) *pPZIP3Δ1::GUS*; (**C**) *pPZIP3Δ2::GUS*; (**D**) *pPZIP3Δ3::GUS*; (**E**) *pPZIP3Δ4::GUS;* and (**F**) *pPZIP3Δ5::GUS*. *pt* (petals), *sp* (sepals), *cp* (carpel), *st* (stamen), and *sg* (stigma). Scale bars: 500 μm.

The results obtained from transgenic plants using the different methodologies indicated in Section 4 and Figure S1 demonstrate that CArG box 1 and CArG box 2 sites are activated by transcription factors. In Figure 3, the histochemical staining shows *A. thaliana* flowers transformed with fragments *pPZIP3Δ1* (complete fragment), *pPZIP3Δ2* (small fragment that contains only CArG box 2), *pPZIP3Δ3* (complete fragment that presents only CArG box 2 since CArG box 1 was deleted), *pPZIP3Δ4* (complete fragment that only presents CArG box 1 since CArG box 2 was deleted), and *pPZIP3Δ5* (complete fragment in which both CArG boxes were deleted), which are represented in Figure S1. Histochemical staining presented in each of the images in Figure 3 was confirmed in three independent transgenic lines, but only representative results from a single line are shown.

Figure 3B shows an open flower of *A. thaliana* transformed with *pPZIP3Δ1*; the four floral whorls can be clearly observed, in which a clear blue staining is apparent, being more marked in the sepals, petals, and carpel. Figure 3C represents an open flower of *A. thaliana*, transformed with *pPZIP3Δ2*, whose fragment only contains CArG box 2; in the image, the blue staining is observed only in sepals and petals with a lower staining than *pPZIP3Δ1*.

**Figure 4:**
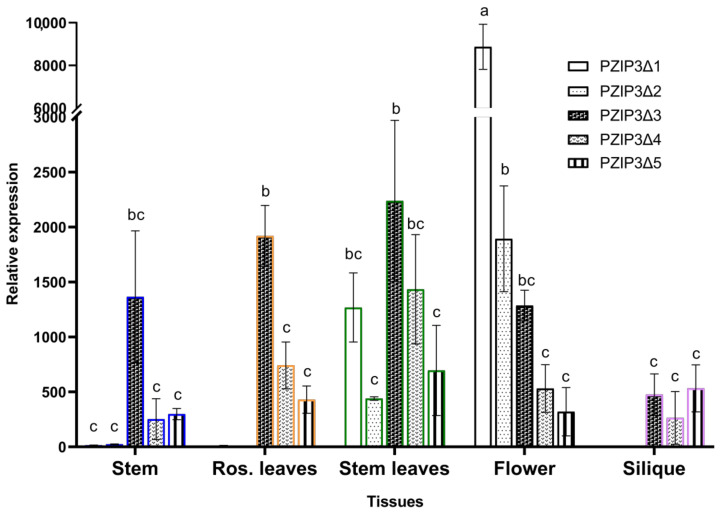
Expression analysis of *GUS* gene via qRT-PCR with constructs *pPZIP3Δ1::GUS*, *pPZIP3Δ2::GUS*, *pPZIP3Δ3::GUS*, *pPZIP3Δ4::GUS,* and *pPZIP3Δ5::GUS* in different *A. thaliana* tissues. Analysis of *GUS* expression was normalized with expression levels of *F-box* and *UBQ10*. *GUS* expression levels in transgenic plants were compared with *GUS* expression levels from a wild-type plant. Data represent the mean ± SD (*n* = 3). Different letters indicate significant differences between the control and samples at each sampling time (*p* < 0.05).

The results of GUS histochemical staining using fragments with site-directed deletions (*pPZIP3Δ3*, *pPZIP3Δ4,* and *pPZIP3Δ5*) appear in Figure 3D–F; these images show a change in staining pattern when compared with Figure 3B,C. Figure 3D,E show a distinctive pattern with blue coloration in sepals, stamens, and stigma, with the coloration being slightly more intense in 3D. This difference in intensity of coloration in floral tissue is observed in the measurement of GUS expression via qRT-PCR (Figure 4); in Figure 3F no blue coloration is observed.

Plants transformed with the construct *pPZIP3Δ3::GUS*, which deleted the CArG box 1, are shown in Figure 3D. Comparing this image with 3B, a loss of coloration in petals and largely of the carpel can be observed, implying a partial functionality on the part of the promoter since only CArG box 2 would be working. Figure 3E corresponds to the transformation with the *pPZIP3Δ4::GUS* construct, which has CArG box 2 deleted; as the spatial staining pattern is similar to that presented in Figure 3D, the result also suggests a partial functionality of the promoter containing only CArG box 1. Figure 3F displays a flower that has undergone transformation with a promoter wherein both CArG boxes have been removed. This observation indicates a diminished functionality of the promoter, as the absence of functional CArG boxes renders it incapable of being activated by MADS transcription factors.

A comparison of GUS expression levels between different images in Figure 3 can be seen in the qRT-PCR analysis of Figure 4. When comparing the GUS expression levels between *pPZIP3Δ1* and *pPZIP3Δ2* (Figure 4), similar expression levels are observed in vegetative tissue, but in floral tissue, the expression is higher in *pPZIP3Δ1*, which is the fragment that contains both CArG boxes; this result suggests the presence of transcription factors that are only found in floral tissue capable of activating the *VviZIP3* promoter.

Comparing the expression levels of all fragments in floral tissue (Figure 4), it can be observed that the *pPZIP3Δ1* fragment, which contains both CArG boxes, is the one that presents the highest degree of expression compared with the other tissues analyzed (Figure 4). Finally, as CArG boxes are removed, it is observed that the *pPZIP3Δ5* fragment is the one with the lowest expression level.

### 2.5. The Functionality of CArG Boxes Present in VviZIP3 Promoter Is Carried out by MADS Proteins

**Figure 5:**
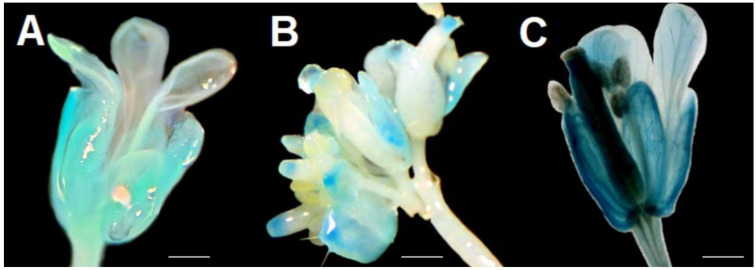
GUS histochemical staining in *A. thaliana* flowers containing the *pPZIP3Δ1::GUS* fragment. (**A**) ag mutant; (**B**) ap1 mutant; and (**C**) wild-type. Scale bars: 500 μm.

**Figure 6:**
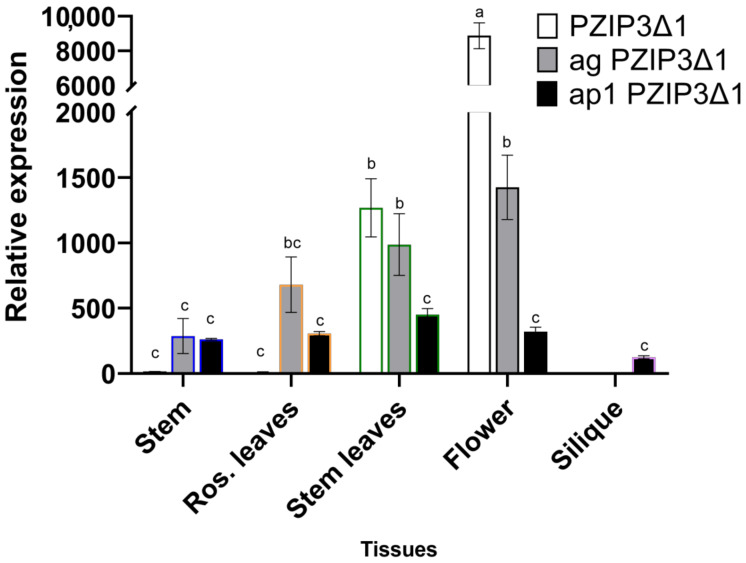
*GUS* gene expression analysis via qRT-PCR in wild-type (white bars), *ag* (grey bars), and *ap1* (black bars) mutants containing the *pPZIP3Δ1::GUS* construct in different *A. thaliana* tissues. Analysis of *GUS* expression was normalized with expression levels of *F-box* and *UBQ10*. *GUS* expression levels in transgenic plants were compared with *GUS* expression levels from a wild-type plant. Data represent the mean ± SD (*n* = 3). Different letters indicate significant differences between the control and samples at each sampling time (*p* < 0.05).

*Agrobacterium tumefaciens* (*A. tumefaciens*) cells transformed with construct *pPZIP3Δ1::GUS* (complete fragment) were used to transform both mutant lines ag and ap1 of *A. thaliana*. Two transgenic lines were obtained for ap1, and one transgenic line was obtained for ag. GUS reporter gene expression was evaluated using histochemical staining (Figure 5) and via qRT-PCR (Figure 6).

Figure 5A corresponds to a flower of an ag mutant that does not have carpels or stamens. This mutant line transformed with the *pPZIP3Δ1::GUS* fragment shows that blue coloration is slightly noticeable in sepals and to a lesser degree in petals.

Figure 5B corresponds to a flower of an ap1 mutant transformed with the *pPZIP3Δ1::GUS* fragment that did not show petals and instead of sepals presents bracts which is a structure closer to a leaf. The blue coloration is slightly noticeable on the bracts and the stigmas of carpels. By comparing the staining level in Figure 5A,B with Figure 3B, the intensity of blue staining is lower; this appreciation is corroborated when comparing GUS expression levels in flowers between Figure 4 and Figure 6.

The expression profile showed a significantly higher level of GUS in the ag mutant compared with the ap1 mutant (Figure 6). This observation is more noticeable by comparing the expression in floral tissue (Figure 6).

The analysis of GUS expression in floral tissue in plants transformed with the *pPZIP3Δ1* fragment showed a lower expression in the ag and ap1 mutant plants compared with the wild-type plants (Figure 6).

## 3. Discussion

There are several mechanisms of regulation of gene expression, which participate at different levels: chromatin level, transcriptional level, post-transcriptional level, translational level, and post-translational level [42]. The specific recognition of response elements by transcription factors and their assembly [43,44,45] in promoters and enhancers is crucial for the beginning of a functional and specific transcription of a gene [46].

On the other hand, different proteins that would be responsible for the transport of Zn in plants have been characterized. Cation diffuser facilitators (CDFs), also called MTPs (“Metal Tolerance Proteins” or “Metal Transport Proteins”) [47], are a group of transporters that allow sequestration in compartments such as the vacuole, acquiring greater relevance in the presence of an excess of Zn [48]. The heavy metal ATPase (“HMA”) [49] are those that would allow the exit of Zn to vascular tissues for the transport of this element through the xylem towards the aerial parts of the plant [50]. CAX-type transporters (cation/proton exchangers) are a family also identified in plants [51]. CAX transporters operate with a cation/H^+^ divalent anti-support mechanism and are localized in the vacuole [14]. Members of the CAX family were first studied to transport Ca^2+^, but additional studies revealed their ability to transport many ions including Zn [52]. Another family of proteins involved in the transport of Zn are the PCR-type transporters (Plant Cadmium Resistance), which have the function of maintaining an optimal concentration of Zn in the roots, acting as a secondary transporter mainly in epidermal cells and the xylem of new roots [53]. One of the most important transporters is the ZIP family (zinc-regulated transporters and iron-regulated transporter-like proteins) [54,55]. ZIP proteins are believed to mediate the majority of Zn uptake from the soil to the cytoplasm of root cells [56]. ZIP proteins have also been shown to be important in plant adaptation to low and fluctuating zinc availability in the soil [57]. Gainza-Cortés et al. [32] performed an expression analysis of different ZIP genes in several tissues and reproductive stages from grapevines showing that *VviZIP3* exhibits a differential expression profile between vegetative and reproductive tissues. The highest *VviZIP3* expression occurred during reproductive development since in the pre-anthesis and flower cluster stages, its expression increased almost fifteen times, suggesting that *VviZIP3* could be more important during the early stages of reproductive tissue development [58,59]. The expression analysis was also carried out in the fruit development stages. Although the *VviZIP3* expression was lower compared with the pre-anthesis and flower stages, the expression was mainly focused on the pericarp, the skin of berries, and a slight activity on seeds. Upon comparing the expression patterns of *VviZIP3* between green parthenocarpic and regular berries during the pre-veraison stage, it became evident that *VviZIP3* was more significantly suppressed in parthenocarpic berries than in their normal counterparts. This observation implies that the expression of *VviZIP3* is essential for the proper development of berries [57]. Gainza-Cortés et al. [32] performed a measurement of the Zn content of different reproductive stages and during several stages of development of berries in the same season. The results showed that the floral state in the anthesis has the highest Zn content. The Zn content is twice as high compared with the pre-anthesis state. As it was determined that the highest Zn content occurs during the floral stage, with a large increase in the *VviZIP3* expression in clusters in pre-anthesis and flowers, then it can be suggested that VviZIP3 is necessary for the transport of the Zn used throughout the reproductive process of the grapevine and especially for the formation of flowers [32]. When comparing the Zn content of normal berries with parthenocarpic berries, it was possible to establish that normal berries have a higher Zn content than parthenocarpic berries [15,60]. When correlating the result of the higher Zn content in normal berries with the result that showed greater *VviZIP3* expression in normal berries, it is suggested that VviZIP3 may be responsible for the fact that the berries can acquire the Zn necessary for their normal development [32].

The in silico analysis of the promoter region of *VviZIP3* enabled the identification of response elements called CArG box, which are related to MADS transcription factors. Computer programs such as Jaspar [38] and ConSite [39] indicated that there was a high probability that the two CArG boxes found in the promoter region of *VviZIP3* were coincident with the consensus sequence CC(A/T)6GG [40] described for MADS transcription factors. The sequence for CArG box 1 is CCTCATTTGG and the sequence for CArG box 2 is ACAAAAATGG, and in both cases, the difference is only one nucleotide with respect to the consensus sequence. Evidence suggests that the binding specificity of a MADS protein primarily arises from the three-dimensional arrangement that monomers undertake to create homodimers, heterodimers, and more complex structures [61]. In this context, there is supporting evidence indicating that the sequence of the CArG box can exhibit variability, and minor deviations within the sequence may not necessarily disrupt the interaction [40,62,63]. One more concern associated with AG and AP1 is that despite their identical nature, AP1 might disrupt the activation of AG target genes and could even potentially take on AG’s role [64]. It is important to highlight that both these transcription factors mutually inhibit each other. Specifically, AP1 is suppressed by SEP3-AG within the inner two whorls, while AG is restrained by SEP3-AP1 within the outer two whorls [65].

In order to try to elucidate a specific gene expression, GUS staining histochemical analysis is one of the most common experiments to perform. The ideal analysis is to isolate the *VviZIP3* promoter, fuse it upstream of the GUS reporter gene, clone it into a vector, and finally transform it into *V. vinifera*. At the time of carrying out this work, there was no efficient protocol for the transformation of grapevines, nor was our work with a *V. vinifera* cultivar able to be transformed [66]. It is for this reason that the methodology of transforming the promoter region of *VviZIP3* into *A. thaliana* was proposed, which allowed us to use transformation protocols [67,68,69] already tested in our laboratory. A problem of this methodology is that the CArG box present in the *VviZIP3* promoter is recognized by the MADS of *V. vinifera* but not by that of *A. thaliana*. It is for this reason that it was necessary to compare the sequences of the MADS proteins of both species. According to the in silico analysis in the search for response elements, it was necessary that AG and AP1 were quite similar, but it was found that other proteins were also classified within very similar clades, among them Apetalla 3 (AP3), Pistillata (PI), Fruitfull (FUL), Sepallata 1 (SEP1), and Sepallata 3 (SEP3) [41]. These analyses reveal that MADS protein sequences are remarkably conserved in both species, and there is a diverse background that MADS proteins are conserved in different species, including domains [70]. Therefore, if the CArG box sequences present in the *VviZIP3* promoter are recognized by MADS proteins from *V. vinifera*, it is likely that they are also recognized by MADS proteins from *A. thaliana*. The results presented in Figure 3, Figure 4, Figure 5 and Figure 6 suggest functionality of the CArG boxes present in the *VviZIP3* promoter.

Comparing Figure 3B,C, differences in staining are observed, with a loss of staining of the carpel and stamens in Figure 3C. Figure 3B represents an open flower of *A. thaliana* transformed with *pPZIP3Δ1* (complete fragment), which is the fragment that contains both CArG boxes. Figure 3C represents an open flower of *A. thaliana*, transformed with *pPZIP3Δ2* (the smallest fragment containing only CArG box 2); the image shows blue staining only in sepals and petals with a lower staining level than *pPZIP3Δ1*. This difference in staining shows that the lack of CArG box 1 modifies the spatiality and intensity of the staining. It is probable that the recognition of CArG box 2 is by AP1 since when a single staining appears in sepals and petals, it would indicate the effect of some gene that is being expressed in that location. A possible participation of AP3 cannot be ruled out due to it having a functionality similar to AP1 or another MADS protein since MADS proteins show a high percentage of identity between them, as well as the fact that the specificity is a result of the tetrameric structure that is able to form between MADS proteins [71], in addition to the importance of AP3 in the formation of petals and stamens in whorls 2 and 3.

Histochemical staining of *A. thaliana* flowers observed in Figure 3D,E represent the site-directed deletions of CArG boxes present in the *VviZIP3* promoter. Figure 3D corresponds to the transformation with the *pPZIP3Δ3::GUS* construct, which is the complete fragment that does not contain the CArG box 1. When comparing this image with 3B, it is observed that there is a loss of blue coloration in petals and the large carpel portion, implying a partial functionality on the promoter since only CArG box 2 would be working. This result is similar to Figure 3C, which also contains CArG box 2 and in which there is no staining of the carpel, although staining does appear in stamens. This result can be explained because even though the *pPZIP3Δ2* and *pPZIP3Δ3* fragments only present CArG box 2, the size of both fragments is different, where *pPZIP3Δ2* contains extra nucleotides that *pPZIP3Δ3* does not have and that could influence the GUS expression. Furthermore, it is not possible to ensure that the same transcription factor is binding to CArG box 2 in both of the fragments compared; it is also a possibility, as previously indicated, that the tetrameric structure is different, generating a different staining pattern. Figure 3E corresponds to the transformation with the *pPZIP3Δ4* construct, which has CArG box 2 deleted. As the spatial pattern of staining is similar in Figure 3D and despite the existence of nucleotide variations between both CArG boxes, the result also suggests a partial functionality of the promoter containing only CArG box 1 and a possible activation of CArG box 1 with the same tetrameric configuration as observed with construct *pPZIP3Δ3* (Figure 3D). Figure 3F represents an *A. thaliana* flower transformed with the *pPZIP3Δ5* construct whose *VviZIP3* promoter contains both deleted CArG boxes; the histochemical staining shows an absence of GUS staining. This result suggests a loss of promoter functionality since neither of the two CArG boxes would be functional and, therefore, the promoter cannot be activated by MADS transcription factors.

Histochemical analysis on two mutants of *A. thaliana* plants for MADS transcription factors (Figure 5) shows a decrease in GUS staining (compared with Figure 3B). These plants do not have these transcription factors. Therefore, they could not form the tetramer and activate the CArG boxes in *pPZIP3Δ1*. Interestingly, MADS transcription factors regulate other MADS transcription factors and allow tetramers to be formed with other MADS transcription factors. In the case of AG, studies show that AG alone is not able to auto-regulate its expression, but together with SEP3 performs positive feedback, regulating AP3, AG, and SEP3 [72], while SEP3 is able to regulate AP1, AP3, and SEP3 [70]. Therefore, a component of the tetrameric complexes such as AG, which participate in whorls 3 and 4, and SEP3, a component of tetrameric complexes that participate in whorls 2–4, are involved in direct and positive regulation of their own tetrameric complexes, in addition to repressing other genes such as AGL24, SVP, and SOC1 [73]. Finally, AG ends up altering the stability of the ABCE system [74]. This alteration of different MADS proteins could affect the activation of CArG box in the promoter region of *VviZIP3*, and demonstrate the scarcity of the staining shown in Figure 5A compared with the images in Figure 3. In relation to AP1, this gene promotes early floral meristem identity in conjunction with LEAFY [75]. Later, it is required for the transition from an inflorescence meristem to a floral meristem and is essential for the normal development of sepals and petals in flowers. If AP1 can activate LEAFY [76] and indirectly SEP3 [75], then an AP1 mutation also ends up affecting almost the entire stability of the ABCE system. Therefore, both MADS transcription factors are affected, altering the activation of the CArG box and, therefore, decreasing the level of staining presented in Figure 5B. Although the antagonistic interaction between AG and AP1 during the development of floral organ identity may help to explain some of the results of GUS staining patterns, it is also important to consider that *VviZIP3* activation occurs after the development of the floral organs, the involvement of additional factors such as the SEUSS-LEUNIG (SEU-LUG) co-repressor complex and SEP3 is essential, and their expression must occur with precision in both temporal and spatial contexts [77].

Contrasting GUS staining in Figure 5A,B with Figure 3B, the intensity of blue staining is lower; this appreciation is corroborated by comparing GUS expression levels in floral tissue in Figure 4 and Figure 6. Comparing GUS expression between *pPZIP3Δ1* and other constructs (Figure 4), similar expression levels are observed in vegetative tissue, except for *pPZIP3Δ3*, which has high expression levels in vegetative tissue, although lower than the expression of *pPZIP3Δ1* in floral tissue. This situation may be the result of a greater number of copies of the foreign DNA being transformed or due to the site where the integration of the foreign DNA occurs; these factors may alter the level of staining [78,79]. The work carried out by Tilly et al. [80] also demonstrated that mutations of a CArG box present in the AP3 promoter of *A. thaliana* generated an increase in the level of reporter gene activity during early stages of flowering, which were not the ones they were mainly studying. Therefore, it is possible to speculate that the deletion made to CArG box 1 could have affected some repressor of vegetative tissue, generating an increase in GUS activity in vegetative tissue. Regarding the expression in floral tissue, the highest level of expression occurs with the *pPZIP3Δ1* fragment that contains both CArG boxes. The *pPZIP3Δ5* fragment, which does not contain a CArG box, shows a lower level of GUS expression, a result that suggests the presence of transcription factors that are found only in floral tissue and that can activate the *VviZIP3* promoter.

When comparing relative expression levels between Figure 5A,B, a higher expression level can be observed in the ag mutant, being more visible in floral tissue. This suggests that AG would have a lower incidence than AP1 in activation of the transgenic promoter.

This difference in the effects of GUS expression by the different CArG boxes present in the *VviZIP3* promoter could be observed in a similar result in the AP3 promoter of *A. thaliana*. AP3 has three CArG boxes in its promoter sequence. Mutations in different CArG boxes generated changes in the expression patterns of the different floral tissues [81].

**Figure 7:**
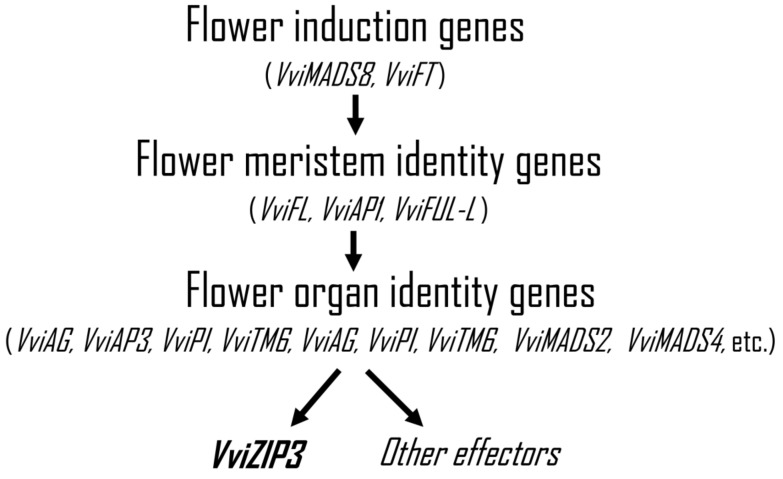
Summary of the proposed involvement of *VviZIP3* in the floral development of grapevines. The sequential steps of molecular control of flowering and some examples of characterized genes that are involved in this process are shown.

Finally, we summarized the current knowledge about the molecular control of flowering in grapevine. Flower formation occurs through a series of sequential steps under strict genetic control [82,83,84]. To initiate the floral transition, the activity of the floral induction genes is necessary; later, the second genes to participate are the floral meristem identity genes, which, in response to different environmental and developmental signals, allow the switch from vegetative to floral state. Third, the floral meristem is patterned in the whorls of the organ primordia through the activity of floral organ identity genes. Fourth, several tissues that constitute the different floral structures are activated by floral organ identity genes [85]. In this last group, we propose that *VviZIP3* is among the numerous downstream effectors that are activated by these genes, whose increased expression would allow a greater entry of Zn during flowering, a stage where the Zn content is critical (Figure 7). Examples of distinctive genes involved in this process include *VviMADS8* [37,83,85], *VviFL* [83], *VviFT* [86], *VviAP1* [37,87], *VviFUL*-L [37,87], *VviAP3* [88,89], *VviPI* [88,89], *VviTM6* [89], *VviAG* [35], *VviMADS2* [36], and *VviMADS4* [36].

## 4. Materials and Methods

### 4.1. Plant Material

The *VviZIP3* promoter was obtained from genomic DNA extracted from grapevine leaves (*V. vinifera* L. Cv. Carménère). The samples were obtained via clonal propagation of the CTVV13 accession, 2015 season, grown at Viña Casa Silva, Los Lingues, San Fernando (34°30′12.9″ S, 70°53′47.8″ W). Expression analyses were performed in *A. thaliana* ecotype Columbia (Col-0) plants and in ag and ap1 mutants of *A. thaliana* ecotype Landsberg erecta (Ler). The mutant seeds were provided by Dr. Aurelio Gómez-Cardenas of the Universitat Jaume I. The ag seeds, deficient in the transcription factor AG, were heterozygous for the mutation, whereas ap1 seeds, deficient in the AP1 transcription factor, were homozygous for their mutation. The tissues analyzed were stem, stem leaves, rosette leaves, flower, and silique.

### 4.2. Purification of Genomic DNA from V. vinifera cv. Carménère

DNA extraction was carried out according to the protocol proposed by Hanania et al. [90] for grapevines. The integrity of the DNA was examined via an agarose gel electrophoresis and purity was examined using an absorbance ratio at 260/280 nm wavelength. The nucleic acid concentration was determined using a spectrophotometer (Infinite M200 PRO, Tecan Trading AG, Mannedorf, Switzerland).

### 4.3. In Silico Analysis of the Cis-Regulatory Regions of the VviZIP3 Promoter Region

The search for cis-regulatory elements in the *VviZIP3* promoter was carried out mainly using the software JASPAR (https://jaspar.genereg.net) accessed on 15 August 2023 [38] and ConSite [39]. Additionally, the databases PLACE (https://www.dna.affrc.go.jp/PLACE/?action=newplace) accessed on 10 June 2018 [91] and PlantCare (bioinformatics.psb.ugent.be/webtools/plantcare/html/) accessed on 10 June 2018 [92] were used, which also have search software for *cis*-regulatory elements.

### 4.4. VviZIP3 Promoter Region Isolation

A search was carried out in the promoter region of *VviZIP3*, consisting of approximately 2000 base pairs upstream of the transcription start site, in the genome of the grapevine cv. Pinot Noir, located in the GENOSCOPE database (www.genoscope.cns.fr/externe/GenomeBrowser/Vitis/) accessed on 20 January 2018. Once this sequence was identified, we proceeded to design the ZPR1 (HindIII-Fw: 5′AAAGCTTCCATGTGTTGAAGAAGTACG3′; NcoI-Rv: 5′TCCATGGGGCTTAAGATGGAGAGTG3′) and ZPR2 (HindIII-Fw: 5′TAAGCTTTATAGATTCCCTCCATACACCC3′; NcoI-Rv: 5′TCCATGGGGCTTAAGATGGAGAGTG3′) primers (Figure S1) using the Primer3 software accessed on 25 January 2018 [93]. With the primers ZPR1-HindIII-fw and ZPR1-NcoI-rv, the PZIP3Δ1 fragment of 1253 bp was amplified. With the primers ZPR2-HindIII-fw and ZPR2-NcoI-rv, the 880 bp PZIP3Δ2 fragment was amplified (Figure S1). Genomic DNA was extracted from *V. vinifera* cv. Carménère to be used as a template for the PCR reaction. An amount of 100 ng of DNA was used in each PCR reaction. An amount of 1.5 mM MgCl_2_, 1× PCR Buffer, 250 nM of each primer, 200 nM dNTPs, and 0.25 U of Platinum^®®^ Taq DNA Polymerase High Fidelity (Invitrogen, USA) was used. The parameters of each PCR were as follows: an initial denaturation step at 95 °C for 10 min, 35 cycles of 95 °C for 45 s, 55 °C for 45 s, and 72 °C for 2 min, and finally 72 °C for 7 min. The PCR products were separated on a 1% agarose gel electrophoresis in 1× TAE buffer. The corresponding bands were cut with a scalpel and the DNA was extracted from the gel using the EZNA Elution Gel Kit system (Omega Bio-Tek, Norcross, GA, USA). The products obtained were checked on a 1% TAE 1× agarose gel electrophoresis and subsequently ligated to the vector pGEMT-Easy (Promega, Madison, WI, USA). The constructs in the vectors, now called *pPZIP3∆1* and *pPZIP3∆2* (Figure S1), were transformed into E. coli for their multiplication. After the vectors were extracted, samples were prepared to determine the nucleotide sequence of *pPZIP3∆1* and *pPZIP3∆2* in Carménère. The sequence of *pPZIP3∆1* and *pPZIP3∆2* in the recombinant plasmids was determined through the sequencing service of the company MACROGEN Inc. (Seoul, Korea).

### 4.5. Construction of VviZIP3 Reporter Gene-Promoter Fusions

Plasmid DNA was subjected to double digestion with restriction enzymes HindIII (Promega) and NcoI (New England Biolabs, Ipswich, MA, USA). An amount of 2 µg/µL of plasmid DNA, 20 u/µL of each restriction enzyme, 8 µL of Buffer E, and 53 µL of sterile and deionized water were used in the reaction. Digestion was carried out at 37 °C for 2 h. The digestion products were separated on a 1% agarose gel electrophoresis, checking the size of the fragments. Subsequently, the PZIP3Δ1 and PZIP3Δ2 fragments were ligated to the binary vector pCAMBIA 1303 (CambiaLabs, Canberra, Australia). For the ligation reaction, approximately 200 ng of vector, 35 ng of the DNA to be inserted, 2 µL of 10× buffer, 2 units of T4 DNA Ligase (Promega), and 4.4 µL of water were used, totaling 20 µL. The reaction was carried out at 4 °C overnight. Previously, the vector pCAMBIA 1303 was subjected to the same digestion with restriction enzymes described previously; in this way, the ligation reaction replaced the CaMV 35S promoter of the vector with PZIP3Δ1 and PZIP3Δ2 fragments. The correct insertion of both fragments was verified via PCR and a 1% agarose gel electrophoresis. The binary vector pCAMBIA 1303 contains both reporter genes GUS and mgfp5; in this way, the *PZIP3::GUS* fusion was obtained, generating new constructs that were called *pPZIP3Δ1::GUS* and *pPZIP3Δ2::GUS*. Once the size of both constructs was confirmed via both PCR and a 1% agarose gel electrophoresis, each vector was transformed into chemically competent bacteria belonging to the *A. tumefaciens* strain GV3101 according to the Gateway^®®^ TOPO vector System methodology (Invitrogen Co., Ltd.) [94].

### 4.6. Transformation of PZIP3 Fragments in A. thaliana

With the *A. tumefaciens* strains containing the constructs *pPZIP3Δ1::GUS* and *pPZIP3Δ2::GUS*, *A. thaliana* Col-0 plants were transformed using floral dipping [67] modified according to [68]. After obtaining the seeds, transgenic plants were selected in a medium containing 25 mg/L of hygromycin [95], obtaining various independent lines of T1 generation.

In order to confirm the transgenesis, genomic DNA extraction was performed from each of the hygromycin-selected plants. With the DNA, a 1% agarose gel electrophoresis was performed on the PCR product, using the primers that amplified each insert of pPZIP3. After verifying that the T1 plants were transgenic, they were analyzed to determine the expression of the reporter gene.

### 4.7. Histochemical GUS Staining

GUS histochemical staining [78] was performed following the protocol of Tapia et al. [96] in tissues of basal leaves, stem leaves, stem, and flowers of three lines belonging to each T1 generation of *pPZIP3Δ1::GUS* and *pPZIP3Δ2::GUS*. The tissues were incubated for 5 h at 37 °C in a substrate solution (100 mM phosphate buffer, pH 7.0, 0.5 mM potassium ferricyanide, K_3_Fe(CN)_6_; 0.1% Triton X-100, 10 mM EDTA, 0.5 mM β-mercaptoethanol, and 1 mg/mL X-Gluc) (PhytoTechnology Laboratories, Shawnee Mission, KS, USA). After staining, the tissues were fixed for 15 min in an FAA solution (85 mL sterile water, 85 mL absolute ethanol, 10 mL glacial acetic acid, and 20 mL 37% formalin). Subsequently, they were bleached in a methanol/acetone solution (3:1) to remove chlorophyll and other pigments and preserved in 75% ethanol. Later, the tissues were visualized in an optical microscope (iScope, Euromex, Arnhem, The Netherlands). As a control, wild-type plants transformed with the vector pCambia 1303 containing the GUS reporter gene under the direction of the CaMV 35S promoter were evaluated; incubation with the substrate was conducted for 5 h.

### 4.8. Analysis of Reporter Gene Expression via Quantitative RT-PCR

Duplicate samples (biological replicates) of basal leaf, stem leaf, stem, and flower tissues were collected from three transgenic T1 lines, used in histochemical staining, and frozen in liquid nitrogen. The samples were processed to extract total RNA using the SV Total RNA Isolation System (Promega) methodology, which is described by the manufacturer. Total RNA integrity was electrophoretically corroborated via formaldehyde agarose gel electrophoresis and their purity via OD260/280 ratio > 1.95. Nucleic acid concentration was determined using an Infinite M200 PRO spectrophotometer (Tecan). After treating the total RNAs with the commercial kit “DNAse Turbo” (Invitrogen) according to the manufacturer’s instructions and eliminating the DNA remains, the synthesis of the first strand of the cDNAs from RNA was carried out for each sampled tissue. For this, the Maxima Reverse Transcriptase kit (Thermo Fisher Scientific, Waltham, MA, USA) was used following the manufacturer’s instructions and, finally, all samples were diluted to a concentration of 25 ng/µL.

Real-time PCR cDNA amplifications were carried out in an Mx3000P QPCR Systems thermal cycler (Stratagene, San Diego, CA, USA). Maxima SYBR Green/Fluorescein qPCR Master Mix (2×) (Thermo Fisher Scientific) was used in all reactions according to the protocol described by the manufacturer. For each of the two biological replicates, real-time PCR reactions were performed in duplicate (technical replicates) for each tissue studied, using 10 µL of Maxima SYBR Green/Fluorescein qPCR Master Mix (2×), 250 nM of each primer, 25 ng/µL cDNA, and nuclease-free water to a final reaction volume of 20 µL. The detection of GUS gene expression was carried out using the specific primers GUS-QF-fw (Fw: 5′-CCTTCTCTGCCGTTTCCAAATCG-3′) and GUS-QF-rv (Rv: 5′-TCACCTGCGTCAATGTAATGTTCTG-3′). qPCR parameters used were an initial denaturation step at 95 °C for 5 min, 40 cycles of 95 °C for 15 s, 55 °C for 17 s, and 72 °C for 20 s, and finally 55 °C for 10 s and 90 °C for 5 s. Fluorescence was measured at the end of each amplification step. The data obtained were manually analyzed and the expression was normalized to F-box gene (NCBI RefSeq NM_112532.1) (Fw: 5′-TTTCGGCTGAGAGGTTCGAGT-3′ and Rv: 5′-GATTCCAAGACGTAAAGCAGATCA-3′) and UBQ10 gene (Polyubiquitin 10, NCBI RefSeq NM_001084884.5) (Fw: 5′-GGCCTTGTATAATCCCTGATGAATAAG-3′ and Rv: 5′-AAAGAGATAACAGGAACGGAAACATAGT-3′) to minimize possible variations in the amounts of templates used. The *F-box* [97] and *UBQ10* [98] genes were selected for normalization of data due to their consistency in levels of transcripts in all tissues studied. The relative amounts of mRNA were calculated using the method described by Pfaffl [99]. *GUS* expression levels in transgenic plants were compared with GUS expression levels from a wild-type plant. Two-way ANOVA was used to analyze statistically significant differences between fragments and different tissues. Subsequently, a Tukey test was performed to compare multiple means, considering significant differences at *p* ≤ 0.05. The analysis was performed with GraphPad Prism 8 software (GraphPad Software Inc., San Diego, CA, USA).

### 4.9. VviZIP3 Promoter Site-Directed Deletions

A site-directed deletion assay was performed on the *PZIP3∆1* fragment for the elimination of the possible binding sites of the MADS-like transcription factors. The Phusion Site-Directed Mutagenesis Kit (Thermo Fisher Scientific, Waltham, MA, USA) was used following the manufacturer’s instructions. In this way, deletions were generated in the *PZIP3∆1* fragment, deleting the CArG boxes and obtaining new fragments of pPZIP3: *PZIP3∆3* does not contain the CArG box 1; *PZIP3∆4* does not contain the CArG box 2 and *PZIP3∆5* in which both CArG boxes were deleted (Figure S1). The *PZIP3∆1* fragment in plasmid DNA was used as a template for the PCR reaction in the Phusion Site-Directed Mutagenesis Kit. In each PCR reaction, 35 µL of sterile water, 10 µL of 5× Phusion HF Buffer, 10 mM dNTPs, 0.5 µM of each primer, 0.02 u/µL of Phusion Hot Start DNA Polymerase, and 1 ng of PZIP3∆1 plasmid DNA were used. Parameters of each PCR were as follows: initial denaturation step at 98 °C for 30 s, 25 cycles of 98 °C for 10 s, 45 °C for 30 s for VvZIP3_AG-fw and VvZIP3_AG-rv primers (Fw: 5′-/5Phos/GTTTTCACCACTTTAATTACTCATA-3′ and Rv: 5′-/5Phos/AATCACTTTTATCAATATTTCGTT-3′), 50 °C for 30 s for VvZIP3_AP1-fw and VvZIP3_AP1-rv primers (Fw: 5′-/5Phos/AGCACCCCATGTGAGGGC-3′ and Rv: 5′-/5Phos/TTATTTGTTAACTACTAATACAAAGGGG-3′), 72 °C for 2 min, and finally 72 °C for 7 min. Products obtained were checked on a 1% TAE 1× agarose gel electrophoresis and, subsequently, a protocol similar to that described above was carried out with T4 DNA Ligase (Promega, Madison, WI, USA) to re-circularize and reconstruct each vector pGEMT-Easy (Promega, Madison, WI, USA). Then, the vectors were isolated to determine the nucleotide sequence of *PZIP3∆3*, *PZIP3∆4*, and *PZIP3∆5* through the commercial sequencing service of MACROGEN Inc. (Seoul, Korea).

### 4.10. Analysis of Reporter Gene Expression in Fragments Obtained from Site-Directed Deletion

Following the same protocol described above, the *PZIP3∆3*, *PZIP3∆4,* and *PZIP3∆5* fragments were inserted into vector pCAMBIA 1303, replacing the CaMV 35S promoter, and thus generating the new constructs *pPZIP3Δ3::GUS*, *pPZIP3Δ4::GUS,* and *pPZIP3Δ5::GUS*. Once the size of the three constructs was confirmed via PCR and 1 × 1% TAE agarose gel electrophoresis, each vector was transformed into chemically competent bacteria belonging to the *A. tumefaciens* strain GV3101. *A. thaliana* Col-0 plants were transformed using flower dipping according to the previously described protocol. After obtaining the seeds, transgenic plants were selected in a medium containing 25 mg/L hygromycin, obtaining various independent lines of T1 generation for each construct. Each of these transgenic plants was checked by extracting genomic DNA, performing PCR and, subsequently, an agarose gel electrophoresis. Afterward, *GUS* gene expression was analyzed in tissues of basal leaves, stem leaves, stem, flowers, and silique via histochemical staining and qRT-PCR in three lines belonging to each T1 generation of *pPZIP3Δ3::GUS*, *pPZIP3Δ4::GUS,* and *pPZIP3Δ5::GUS*, following the same protocols mentioned above.

### 4.11. Transformation of Mutant Lines of A. thaliana

*GUS* reporter gene expression, directed by the *VviZIP3* promoter, was evaluated in plants of mutant lines ag and ap1 of *A. thaliana* ecotype Ler-0. The ag seeds used, deficient in transcription factor AG, were heterozygous for the mutation; whereas the ap1 seeds used, deficient in AP1 transcription factor, were homozygous for their mutation. *A. tumefaciens* cells, transformed with *pPZIP3Δ1::GUS* construct, were used to transform both mutant lines using flower dipping according to the similar methodology previously described. The transgenic plants obtained were selected in a medium containing 25 mg/L of hygromycin, generating various lines independent of T1 generation; PCR of their genomic DNA and agarose gel electrophoresis were performed to verify transgenesis using the protocol described above. For ap1, two transgenic lines were obtained and for ag, one transgenic line was obtained. Using the protocols already described, GUS reporter gene expression was evaluated in tissues of basal leaves, stem leaves, stem, flowers, and silique using histochemical staining and qRT-PCR.

### 4.12. Statistical Analysis

ANOVA was used to analyze statistically significant differences. The normality of the data was confirmed before the ANOVA test. Subsequently, a Tukey test was performed to compare multiple means, considering significant differences at *p* ≤ 0.05. The analysis was performed with GraphPad Prism 8 software (GraphPad Software Inc., San Diego, CA, USA).

## 5. Conclusions

In this work, we characterized the expression profile and the promoter regions of a gene that encodes a Zn transporter involved in the fruit development of *V. vinifera* cv. Carménère. We identified *cis*-regulatory elements in the promoter of *VviZIP3*, which could be recognized by MADS transcription factors, and their expression profile showed that it is mainly expressed in floral tissue. The results strongly suggest that CArG box 2 could play a more important role in the induction of *VviZIP3* expression than CArG box 1.

## Figures and Tables

**Table 1:** Sequences and location of main selected response elements in the *VviZIP3* promoter. The consensus sequence is highlighted in black.

Response Element	Transcription Factor	Predicted Sequence	Distance to Transcription of Start Site (TSS)
AG	MADS	GATT**CCTCATTTGG**GTT	1115
AP1	MADS	TAA**ACAAAAATGG**ACAGCAC	181
WRKY71OS (GARE)	Repressor of the GA signaling pathway	GTAC	346
WRKY71OS (GARE)	Repressor of the GA signaling pathway	GTAC	1521
ABRELATERD1 (ABA)	ABRE (ABA-responsive element)	CACGTGC	832
ABRELATERD1 (ABA)	ABRE (ABA-responsive element)	CAAGT	77
Athb-1	Homeodomain–Leucine Zipper I (HD-Zip I)	AATAATTG	330
Athb-1	Homeodomain–Leucine Zipper I (HD-Zip I)	CAATTATT	111

## Data Availability

Data are contained within the article/Supplementary Materials.

## Supplementary Materials

The following supporting information can be downloaded at: https://www.mdpi.com/article/10.3390/plants12183322/s1, Figure S1: Graphical view of the main fragments obtained from *VviZIP3* promoter.
