# Supplementary figures and images for: Floral Development on Vitis vinifera Is Associated with MADS-Box Transcription Factors through the Transcriptional Regulation of VviZIP3
Source: Plants (Basel). 2023 Sep 20;12(18):3322. doi: 10.3390/plants12183322 (PMC10535425; doi:10.3390/plants12183322)

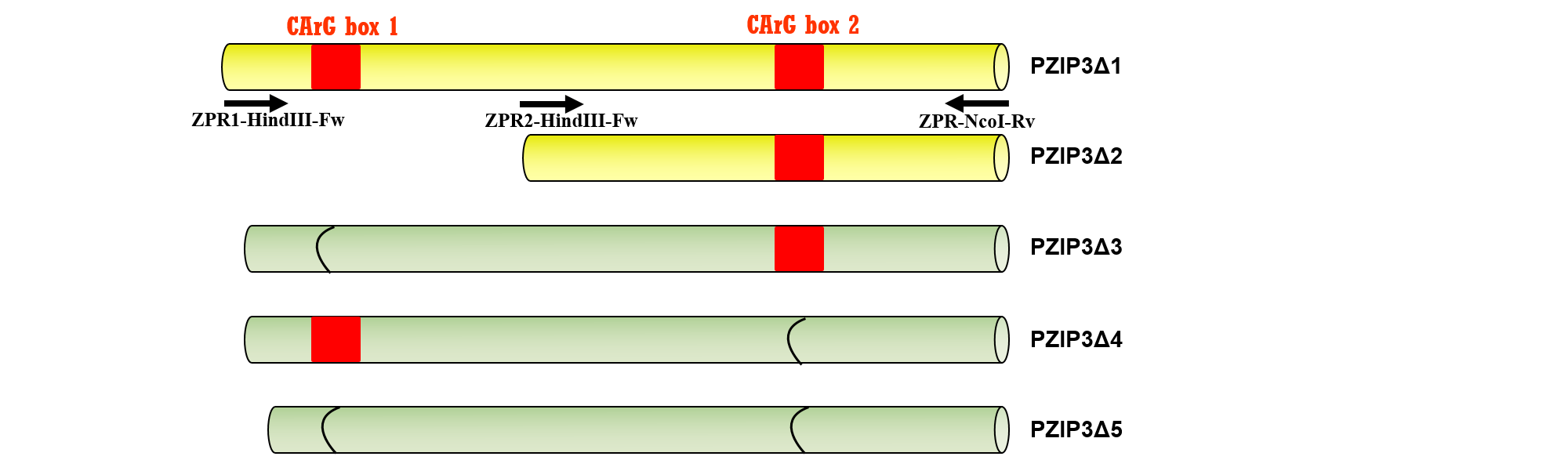

Supplement: Supplementary file 1 [file plants-12-03322-s001.zip › plants-2588834-supplementary.tif]
